# Meningeal Lymphatic and Glymphatic Structures in a Pelagic Delphinid (*Delphinus delphis*)

**DOI:** 10.3390/ani15050729

**Published:** 2025-03-04

**Authors:** Olivia N. Jackson, Tiffany F. Keenan, Nathan P. Nelson-Maney, Sentiel A. Rommel, William A. McLellan, D. Ann Pabst, Alexander M. Costidis, Kathleen M. Caron, Dawn N. Kernagis, David S. Rotstein, Victoria G. Thayer, Craig A. Harms, Marina A. Piscitelli-Doshkov, Paul Doshkov, Lorian E. Schweikert, Kara E. Yopak, Molly Braun, Michael S. Tift

**Affiliations:** 1 Department of Biology and Marine Biology, College of Science and Engineering, University of North Carolina Wilmington, Wilmington, NC 28403, USA; keenant@uncw.edu (T.F.K.); rommels@uncw.edu (S.A.R.); mclellanw@uncw.edu (W.A.M.); pabsta@uncw.edu (D.A.P.); schweikertl@uncw.edu (L.E.S.); yopakk@uncw.edu (K.E.Y.); tiftm@uncw.edu (M.S.T.); 2 Department of Cell Biology and Physiology, School of Medicine, University of North Carolina at Chapel Hill, Chapel Hill, NC 27599, USA; nathan_nelson-maney@med.unc.edu (N.P.N.-M.); kathleen_caron@med.unc.edu (K.M.C.); 3 Marine Mammal Solutions LLC, Norfolk, VA 23502, USA; 4 DEEP, Bristol BS11 8AR, UK; dawn.kernagis@deep.com; 5 Department of Neurosurgery, School of Medicine, University of North Carolina at Chapel Hill, Chapel Hill, NC 27599, USA; 6 Marine Mammal Pathology Services, Olney, MD 28032, USA; drdrot@gmail.com; 7 Center for Marine Science and Technologies, North Carolina State University, Morehead City, NC 28557, USA; vgthayer@ncsu.edu (V.G.T.); caharms@ncsu.edu (C.A.H.); 8 North Carolina Marine Fisheries, Department of Environmental Quality, Morehead City, NC 28557, USA; 9 Department of Clinical Sciences, College of Veterinary Medicine, North Carolina State University, Raleigh, NC 27695, USA; 10 North Carolina Aquariums Jennette’s Pier, Nags Head, NC 27959, USA; marina.doshkov@ncaquariums.com; 11 Cape Hatteras National Seashore, Manteo, NC 27954, USA; paul_doshkov@nps.gov; 12 Department of Neurosurgery, Medical College of Georgia, Augusta University, Augusta, GA 30912, USA; mobraun@augusta.edu

**Keywords:** brain, glymphatic, meningeal lymphatic, marine mammal, cetacean, morphology, anatomy, neuropathology, histochemistry, diving physiology, CSF mechanics

## Abstract

The glymphatic system, an analog of the peripheral lymphatic system in the brain, and the meningeal lymphatic system are critical to central nervous system health. The glymphatic system functions to distribute cerebrospinal fluid and important compounds throughout the brain and to remove metabolic waste. The flow of cerebrospinal fluid through this system is affected by changes in cerebral blood flow, intracranial pressure, and vascular tone. Cetaceans experience profound cardiorespiratory alterations while diving that can directly affect cerebrospinal fluid and blood flow and, thus, glymphatic function. Our goal was to investigate glymphatic and lymphatic system structures, including perivascular spaces, aquaporin-4 water channels, meningeal lymphatic, and dural venous sinus vessels in the common dolphin (*Delphinus delphis*), using immunofluorescent labeling, histochemical staining, and postmortem computed tomography (CT) angiography. We highlight perivascular spaces and aquaporin-4 water channels surrounding blood vessels in the parenchyma and demonstrate evidence of meningeal lymphatic vessels and associated dural venous sinuses. These results demonstrate that common dolphins possess the key anatomical structures required for functional glymphatic and meningeal lymphatic systems. Future studies can build upon these anatomical discoveries to study the function and role of these systems in brain health in this species.

## Simple Summary

The meningeal lymphatic and brain glymphatic systems play crucial roles in the maintenance of brain health through the distribution of fluids and the removal of waste products. This system was recently described for the first time in a wild mammal, the bottlenose dolphin (*Tursiops truncatus*). Our study focuses on the meningeal lymphatic and brain glymphatic systems in a deeper-diving offshore species, the common dolphin (*Delphinus delphis*). We examined brain tissues using computed tomography (CT), histochemical techniques, and immunofluorescent labeling and found that common dolphins possess key structures of the lymphatic and glymphatic systems which are similar to those described in other mammals. This anatomical information in dolphins can provide insights into how pathogens like bacteria and viruses might enter the brain, which could be valuable for understanding central nervous system diseases in marine mammals.

## 1. Introduction

The recently discovered brain glymphatic and meningeal lymphatic systems comprise the glial lymphatic (glymphatic system or perivascular system) and meningeal lymphatic system. The glymphatic system consists of the cerebrospinal fluid (CSF)-filled periarterial compartment surrounding blood vessels, the brain parenchyma, and perivenous pathways, while the meningeal lymphatic system consists of lymphatic vessels within the surrounding dural tissues and the dural venous sinuses [1]. The glymphatic system maintains cerebrospinal fluid (CSF) and interstitial fluid exchange through perivascular spaces surrounding the blood vessels within the parenchyma and is confluent with the subarachnoid space [2]. Rodent studies have demonstrated that glymphatic function is aquaporin-4 (AQP4)-dependent and that the perivascular localization of AQP4 is necessary to facilitate fluid exchange throughout the brain [3]. AQP4 is highly localized to the perivascular endfeet of astrocytes which surround the vasculature throughout the brain. This anatomical organization facilitates the influx of fluid from the perivascular space into the interstitium [3]. AQP4 is also highly expressed along efflux pathways, including surrounding veins and throughout white matter tracts, facilitating the outflow of fluid from the brain [4]. The meningeal lymphatic vessels, located peripherally within the dura, serve as a connection between the perivascular space and the peripheral body [2]. The dural venous sinuses, including the superior sagittal sinus and transverse venous sinus, are a network of large-caliber, thin-walled vessels that contribute to the maintenance of intracranial pressure and serve as the primary drainage route of deoxygenated blood from the brain to the systemic circulation via paired internal jugular veins [5]. These two systems are functionally connected and work in tandem—the glymphatic system exchanges fluids into and out of the parenchyma, while the meningeal lymphatic system works more peripherally and drains fluid and solutes from the intracranial compartment. This anatomical arrangement facilitates the distribution of important compounds and the clearance of waste products from the brain, maintains fluid pressure within the brain, and permits brain immune surveillance [6,7,8,9,10,11,12,13].

Through the use of novel imaging techniques, several physiological drivers have recently been reidentified as important modulators of CSF flow and glymphatic clearance [1]. For example, in rodents, there was a 60% increase in the volume of the interstitial space within brain tissue during both natural sleep and anesthesia, which significantly increased waste clearance [10]. Respiration causes pressure fluctuations in the body that can facilitate CSF efflux along venous and cranial nerve pathways [14]. The cardiac cycle also drives CSF in a pulsatile fashion into the parenchyma along the perivascular space surrounding central nervous system (CNS) blood vessels [15]. Arterial vasomotion, which is largely independent of the cardiac and respiratory cycle, propels intraventricular CSF flow and enhances brain clearance [16]. Dysfunction or impairment of these glymphatic drivers, such as ischemia–reperfusion injuries following stroke or chronic obstructive sleep apnea, can result in altered brain function, neurovascular pathogenesis, neurodegenerative disease, and secondary brain injury [17].

The glymphatic and meningeal lymphatic systems have been described in several terrestrial vertebrates [7,10,18,19,20], suggesting that these systems may be evolutionarily conserved in vertebrates [2]. In addition to these vertebrates, Keenan and colleagues recently described the first evidence of these systems in a wild marine mammal (*Tursiops truncatus*; bottlenose dolphin), including perivascular spaces, astroglial AQP4 water channels, meningeal lymphatic vessels, and dural venous sinus architecture [21]. These results provide evidence for the conservation of glymphatic and meningeal lymphatic system structures in diving mammals, providing the first report in one of the most well-studied marine mammal species.

Marine mammals exhibit a pronounced respiratory sinus arrhythmia. On a dive, they experience sustained bradycardia and peripheral vasoconstriction, and upon surfacing, they exhibit tachycardia and the reperfusion of peripheral tissues, e.g., [22,23,24,25,26]. This dive response is critical to the ability of the animals to maintain mean arterial blood pressure [27]. Thus, changes in respiration, heart rate, and peripheral vasomotion that are known to impact the function of the glymphatic system in terrestrial mammals reviewed by [1], are all routinely experienced by diving mammals [1,10,16,28,29].

Cetaceans also possess a derived cerebral blood supply that could impact the flow of fluids into and out of the brain [30,31,32,33]. While the internal and external carotid arteries are the cerebral blood supply in most terrestrial mammals, cetacean cerebral arterial blood supply is provided by several *retia mirabilia*, which eventually coalesce into the epidural rete [34,35,36,37,38,39,40]. Cetaceans also possess profound adaptations to their cranial morphology, which include the complete remodeling of the nares, loss of olfactory nerves, and a reduction in the ethmoid plate [41,42,43,44,45]. This extreme remodeling of the skull, known as telescoping reviewed by [42,46]), alters the position of the cetacean cerebellum and foramen magnum [46,47,48,49]. This remodeling results in the basal positioning of the transverse venous sinus structures, which differs from the more dorsal position observed in humans and mice [50].

Despite the diving capabilities of both shallow and deep diving cetaceans, investigations of stranded individuals have demonstrated susceptibility to CNS pathologies and hypoxic insults [51,52,53,54,55,56,57,58]. These observations suggest that cetaceans are still vulnerable to diving-related injuries. Additionally, certain species of cetaceans, such as beaked whales and dolphins, including the common dolphin, appear to be susceptible to decompression-like injuries [23,59,60,61], neurotoxins [62], and neuropathological changes (amyloid beta and tau protein accumulation) that are associated with Alzheimer’s disease in humans [58,63]. These CNS vulnerabilities highlight the importance of understanding the presence and characteristics of glymphatic and meningeal lymphatic systems in marine mammals that routinely experience physiological responses associated with diving.

This study investigated the gross macroscopic and microscopic morphology of the dural venous architecture, meningeal lymphatic vessels, perivascular spaces, and astroglial AQP4 water channels in the offshore common dolphin, *Delphinus delphis*. This species provides an opportunity to broaden our understanding of this system within the delphinids, by investigating a closely related species to the previously investigated bottlenose dolphin [21], but one that inhabits a pelagic, rather than a coastal environment. Pelagic delphinids routinely undergo deeper dives than coastal bottlenose dolphins reviewed by [24]). Additionally, the common dolphin is one of the most commonly stranded pelagic delphinid species in North Carolina [64], thus providing access to specimens for anatomic study.

## 2. Materials and Methods

The minimum requirements for a functional terrestrial mammalian glymphatic system includes the production of CSF and the presence of a vascular network within the parenchyma, which is ensheathed by glial cell endfeet lined with AQP4 water channels, which form the perivascular spaces [1]. This system works with meningeal lymphatic vessels positioned along the dural venous sinuses, which, in turn, drain to the peripheral body [2]. To document the glymphatic and meningeal lymphatic systems in the common dolphin (*D. delphis*), this study utilized gross and microscopic techniques.

### 2.1. Specimens

All gross and histological samples included in this study were opportunistically collected from common dolphins (N = 4) that stranded along the Atlantic coast of North Carolina between 2022 and 2023 (Table 1), except for one specimen used for computed tomography (CT) imaging, IFAW-12-364, which stranded in Massachusetts in 2012 and was subsequently frozen. To be included in this study, specimens must have been in fresh condition [Smithsonian Institution (SI) codes 1–2] [65], meaning the animal either live-stranded (code 1) and subsequently expired or was humanely euthanized for reasons unrelated to this study, or was recently deceased with minimal decomposition (code 2). Additionally, a necropsy examination and histopathologic evaluation were conducted to determine sex, life history category, and health status to account for CNS disease and injury [66].

Tissues were prepared using methods previously established by Keenan and colleagues [21]. Briefly, the flensed skull was cross-sectioned with a hand saw just rostral to the sagittal crest. The caudal portion of the skull was immersion fixed in a large volume of 10% Neutral Buffered Formalin for a minimum of two weeks. This method permitted tissues to be fixed in situ within the skull and subsequently subsampled at regions of interest.

### 2.2. Gross Dissection

**Figure 1:**
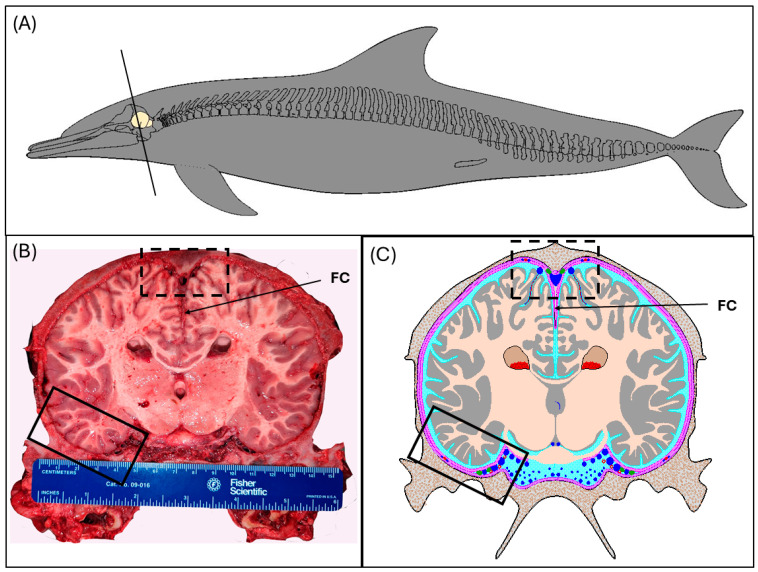
Overview of methods used to gather data and samples for this study. Tissues included were obtained through opportunistic tissue collection from common dolphins (*D. delphis*) that stranded along the coast of North Carolina, except for the specimen used for computed tomography imaging, IFAW-12-364, which was stranded in Massachusetts in 2012. (**A**) Schematic of the common dolphin displaying the position of the brain within the skull and the level at which the section was made (black solid line). The flensed skull was sectioned just rostral to the sagittal crest at the apex of the skull. (**B**) Photograph of the caudal face of the brain section at the position shown in (**A**) (Animal ID: JPIER040). The caudal portion of the skull was immersion fixed and subsequently sampled at the following regions: superior sagittal sinus (dotted black box), transverse venous sinus (solid black box), cerebrum, and cerebellum. Note that the cerebellum is not captured at this level of sectioning and sampling of this tissue was caudal to the section face. (**C**) Schematic of B displaying the brain in relation to the dura (pink and magenta), meningeal lymphatic vessels (green), and cerebrospinal fluid (light blue) within the subarachnoid compartment. The venous vasculature (dark blue) includes both dural venous sinuses, with the relative position of the superior sagittal sinus (dotted black box) and transverse venous sinus (solid black box) displayed. The superior sagittal sinus is formed by the adherence of the periosteal (magenta; outermost layer beneath the calvarium) and meningeal layer of the dura (magenta).

Following adequate fixation of tissues, gross dissection took place targeting routine sites for collection in terrestrial mammals, namely humans and mice, and sites previously examined in Keenan et al. (in review) [21] (Figure 1). These sites included the dural venous sinuses (superior sagittal sinus and transverse venous sinus) and adjacent meningeal tissue to examine meningeal lymphatic vessels. Additionally, parenchymal samples from the cerebrum and cerebellum were taken to examine perivascular spaces and AQP4 water channels. During collection, regions grossly suspected of pre- or perimortem injury or damage were avoided for this study.

For selected individuals, at the gross dissection, digital photo-documentation was used to illustrate the presence and position of the superior sagittal sinus and transverse venous sinus in relation to meningeal and skeletal elements as well as the underlying brain parenchyma. Digital images were used in the creation of anatomical schematics using computer-aided design software (FastCAD-32, Evolution Computing, Inc., Pheonix, AZ, USA).

### 2.3. Computed Tomography

Computed tomography (CT) was paired with gross dissection of a singular fixed specimen (IFAW-12-364; see Table 1) to provide a detailed description of intracranial venous circulatory morphology and to confirm major venous pathways associated with glymphatic and meningeal lymphatic vasculature in terrestrial mammals. This analysis was performed on the head of an intact specimen, which was subsequently sectioned. To prepare the specimen for CT, a vascular purge using 0.9% phosphate-buffered saline solution was flushed through the arteries before injection of contrast media. As the study focused on the cranial venous system, the saline solution was flushed through the arteries, exiting via the veins, until the outflow ran clear. Once this was achieved, the flush was stopped, and the specimen was refrigerated to allow for drainage of the solution.

Veins were prepared by inserting 5 mL balloon catheters until a seal was formed, then introducing a mixture of liquid latex and barium sulfate suspension (Liquid Polibar Plus, E-Z-EM Canada Inc., Montreal, QC, Canada). The specimen was refrigerated for 2 days to allow the mixture to cure, then frozen at −20 °C. Subsequently, CT scanning was performed at 1 mm thickness every 0.2 mm on a Siemens Sensation 16 (Siemens, Munich, Germany) at North Carolina State University College of Veterinary Medicine [35]. DICOM data were post-processed using Amira 6.3.0 software package (FEI Science Visualization Group, Burlington, MA, USA). Images were either orthoslice (direct orthogonal slice data from the scan volume) or shaded-based volume renderings, with no resampling or deconvolution applied.

These CT scans were used to identify areas of interest to target during detailed gross dissections to collect for histologic microscopic investigation.

### 2.4. Histological Preparation

Following gross dissection of brain tissues fixed in situ, meningeal (from the superior sagittal sinus, transverse venous sinus, and adjacent meninges) and perivascular space tissues (from the cerebrum and cerebellum) were cut and oriented within cassettes and placed in 10% Neutral Buffered Formalin. Fixed samples were then embedded in paraffin and serial sectioned between 8 and 15 µm on either a Finesse 325 rotary microtome (Thermo Fisher Scientific Inc., Waltham, MA, USA) or American Optical 820 rotary microtome (American Optical Company, Buffalo, NY, USA). Sections were floated on a 40 °C bath containing distilled water and mounted onto glass Superfrost plus slides (Fisherbrand^TM^ Superfrost^TM^ Plus Microscope Slides, Thermo Fisher Scientific Inc., Waltham, MA, USA) and allowed to dry overnight at room temperature.

### 2.5. Histologic and Fluorescent Immunofluorescent Labeling

To investigate the microscopic morphologic features of meningeal lymphatic vessels and perivascular spaces, standard histological staining was paired with specialized fluorescent techniques that have been established in human and mice models [67,68], as well as another delphinid species (Keenan et al., in review) [21]. Standard histochemical techniques included Harris’ version of Weigert’s Hematoxylin and Eosin (H&E) in 70% ethanol to examine the general anatomy of the dural meningeal lymphatics and the perivascular spaces within the brain parenchyma. Slides were deparaffinized in two changes in toluene for approximately 5 min. Slides were then re-hydrated in descending percentages of ethanol (100%, 100%, 90%, 70%, and 50%) for three minutes, then placed in de-ionized water. After staining in Hematoxylin for 4 min, tissues were rinsed in tap water and placed in de-ionized water for 2 min, followed by Eosin for 1.5 min. Slides were dehydrated through ascending percentages of ethanol (50%, 70%, 90%, 100%, and 100%) for one minute each, then placed in toluene. Coverslips were adhered using Permount (Fisher Chemical^TM^ Permount^TM^ Mounting Medium, Thermo Fisher Scientific Inc., Waltham, MA, USA) and allowed to dry for 24 to 48 h before imaging. Additionally, this study utilized an adapted Masson’s Trichrome (M3) staining protocol previously established by Keenan et al. (2022) for cetacean tissue [69,70] to examine the connective tissue elements of the lymphatic vasculature and surrounding dural tissue. Slides stained with Masson’s Trichrome were de-paraffinized and rehydrated following the methods stated above for Hematoxylin and Eosin staining. Following rehydration steps, slides were stained in Harris’ Hematoxylin (6 min), dipped in picric acid, washed in tap water for 10 min, stained in acid fuchsin (5 min) and Biebrich scarlet (3 min), differentiated in phosphomolybdic acid (5 min), transferred to light green SFS yellow (4 min), and differentiated in acidified water (2 min). Slides were then dehydrated to toluene and cover-slipped using Permount, following the same protocol as above. Images were obtained using a Leica Thunder Imager Tissue microscope (Leica Microsystems CMS GmbH, Wetzlar, Germany), using objectives between 5× and 20× non-oil immersion lenses.

To identify meningeal lymphatic structures, immunofluorescent labeling was conducted and imaged using confocal microscopy. Tissues were prepared for immunofluorescent labeling using the same protocol for histological staining, described above. While previous studies have demonstrated the use of immunohistochemical investigations on formalin-fixed paraffin embedded tissues [57,71], it was unknown whether commercially available antibodies would be appropriate for this species of delphinid. Samples of small intestine and mesenteric lymph node were collected as positive controls to ensure that the antibodies used were targeting lymphatic endothelial cells (see Supplementary Figure S1). Tissue samples were hybridized with antibodies targeting the proteins DAPI/HOESCHT, Prospero-related homeobox-1 (Prox-1), and vascular endothelial growth factor receptor 3 (VEGFR3) [57,71]. Details of all antibodies used (in 1:200 dilution) are in Table 2. Paraffin-embedded sections of meningeal and intestinal tissue were affixed to slides by heating at 60 °C for 60 min. Slides were deparaffinized by the following protocol: two 10 min washes in CitriSolv (CitriSolv™ Hybrid Solvent and Clearing Agent, Thermo Fisher Scientific Inc., Waltham, MA, USA), followed by two 10 min washes in 100% ethanol, then sequential 5 min washes in 95%, 75%, and 50% ethanol, and finally two 5 min washes in phosphate-buffered saline (PBS). Sections were placed in a sodium citrate buffer (10 mM Sodium citrate, 0.5% Tween 20, pH 6.0) and microwaves in a safe pressure cooker at 70% power in a 1000-Watt microwave oven to perform antigen retrieval. The tissue was allowed to cool to room temperature while remaining in the sodium citrate buffer. Slides were then blocked for 4 h in PBS containing 5% normal donkey serum and 0.5% Tween-20. Primary antibodies, diluted in the blocking solution, were applied and incubated overnight at room temperature for 12–16 h. Following primary antibody incubation, slides were washed three times for 5 min each with blocking buffer. Secondary antibodies were added and incubated at room temperature for 4–6 h in the blocking buffer. Nuclei were stained using DAPI for 10 min. Finally, slides were mounted with ProLong Gold antifade (ProLong™ Gold Antifade Mountant, Thermo Fisher Scientific Inc., Waltham, MA, USA) mounting media and covered with a 1 mm thick coverslip. Immunofluorescent imaging was conducted at the Michael Hooker Imaging Core at the University of North Carolina at Chapel Hill using an Olympus SM 800 confocal microscope. Image processing was carried out with Fiji Image J2 software [72].

To identify AQP4 expression, immunofluorescence labeling was performed on paraffin sections and slides were imaged on a Keyence BZ-X810 fluorescence microscope. Slides were deparaffinized in three changes in xylene for 3 min each. Slides were then re-hydrated in descending percentages of ethanol (100%, 100%, 95%, 70%, and 50%) for three minutes each, then placed in de-ionized water. Antigen retrieval was performed in sodium citrate buffer in a steamer for 20 min. Slides were stained with anti-AQP4 (Millipore Sigma AB3594, Sigma-Aldrich Inc., St. Louis, MO, USA) and anti-GFAP (Invitrogen PA5-143587, Thermo Fisher Scientific Inc., Waltham, MA, USA) in 5% normal donkey serum in PBS and 0.3% Triton X overnight at 4 °C. Slides were washed three times for 5 min each in PBS and 0.3% Triton X. Donkey anti-rabbit 488 (Invitrogen A-21206, Thermo Fisher Scientific Inc., Waltham, MA, USA) and donkey anti-goat 680 (Invitrogen A-21084, Thermo Fisher Scientific Inc., Waltham, MA, USA) Alexa Fluor secondaries and Lycopersicon Esculentum (tomato) Lectin Dylight 594 (Invitrogen L32471, Thermo Fisher Scientific Inc., Waltham, MA, USA) were incubated in 5% normal donkey serum in PBS and 0.3% Triton X for 2 h at room temperature. Slides were washed three times for 5 min each in PBS and 0.3% Triton X and mounted with Prolong Diamond Antifade Mountant with DAPI (Invitrogen P36971, Thermo Fisher Scientific Inc., Waltham, MA, USA).

The NCBI Basic Local Alignment Search Tool (BLAST+ 2.16.0) was used to compare protein sequences of field-standard lymphatic endothelial cell markers across multiple species, including bottlenose dolphin (*T. truncatus*), house mouse (*Mus musculus*), human (*Homo sapiens*), chimpanzee (*Pan troglodytes*), bonobo (*Pan paniscus*), and common dolphin (*D. delphis*). This analysis focused on identifying highly conserved genes and epitopes, such as Lyve-1, Prox-1, VEGFR3, and Podoplanin, to assess protein similarity and guide the selection of lymphatic-specific markers for immunofluorescence. The antibodies targeting Prox-1 and VEGFR3 were ultimately chosen due to the highly conserved nature of these proteins across mammals, as determined by their amino acid sequence similarity (see Table 3 for detailed comparisons between humans and common dolphins).

To confirm the functionality of immunofluorescent markers for identifying lymphatic endothelial cells in common dolphin tissues, small intestine samples were collected from a subset of individuals to serve as positive controls. These samples validated that the antibodies specifically targeted lymphatic endothelial cells. Additionally, leptomeningeal tissues were used as negative controls by comparing the same sections of tissue, one labeled with secondary antibodies only to one processed using the full experimental protocol, which included both primary and secondary antibodies. The control studies demonstrated that Prox-1 and VEGFR3 markers produced distinct positive labeling, clearly distinguishable from tissue autofluorescence. The results from the intestinal and meningeal tissues are shown in Supplementary Figures S2 and S3, respectively.

## 3. Results

The results presented here provide anatomical evidence for the presence of the structural components of the glymphatic and meningeal lymphatic systems in an offshore delphinid, the common dolphin (*D. delpis*), including cranial dural venous sinuses and associated meningeal lymphatic vessels, and perivascular spaces surrounded by AQP4 water channels within the brain parenchyma.

### 3.1. Dural Venous Sinuses

**Figure 2:**
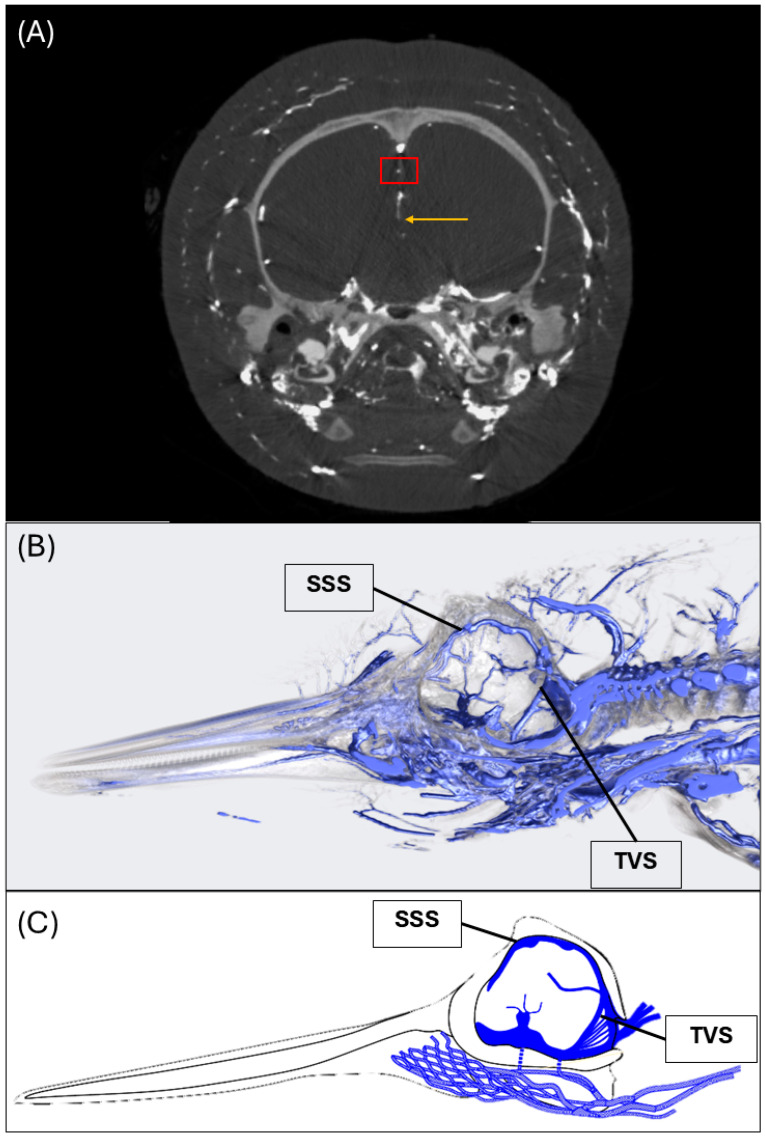
CT angiography digital reconstruction of the common dolphin (*D. delpis*) displaying the key structural features of the superior sagittal and transverse venous sinuses. (**A**,**B**) Animal ID: IFAW12-364. CT angiography of the common dolphin (*D. delpis*) reveals the morphology of the dural venous sinuses and the associated intra- and extra-cranial venous connections. (**A**) A CT orthoslice of the common dolphin in transverse plane at the level of the sagittal crest demonstrating the superior sagittal sinus (red box) and inferior sinus (yellow arrow). (**B**) A CT reconstruction in the mid-sagittal plane demonstrating the position of the superior sagittal sinus (SSS) and transverse venous sinus (TVS). (**C**) Simplified schematic of the dural venous sinuses displaying positions and connections of the superior sagittal sinus (SSS) and transverse sinus (TVS).

The morphology of the intracranial dural venous sinuses was investigated using computed tomography (CT) angiography, detailed gross dissection, and histological examination which are presented in this order below. The CT and digital angiography reconstruction revealed a venous network surrounding the brain (Figure 2). This venous network includes the superior sagittal sinus (Figure 2A), inferior sagittal sinus (Figure 2A), and transverse venous sinus (Figure 2B). The superior sagittal sinus merges with the transverse venous sinus plexus that wraps around the base of the skull (Figure 2B) where venous blood is eventually drained via the internal jugular veins. Additionally, the venous networks in the cervical region and associated extracranial vasculature can be observed in the reconstruction in the sagittal plane (Figure 2B). These results confirm the position and connections of the cranial and extracranial venous vasculature of the common dolphin.

The superior sagittal sinus lies along the dorsal midline within the braincase and is positioned just below the skull within the meninges surrounding the brain, seated directly above the falx cerebri (Figure 1B,C and Figure 2). The superior sagittal sinus joins the transverse venous sinus, located bilaterally surrounding the tentorium cerebelli, through the confluence of the sinuses (Figure 2). Venous blood is ultimately directed out of the skull through the sigmoid sinus, to the internal jugular veins.

**Figure 3:**
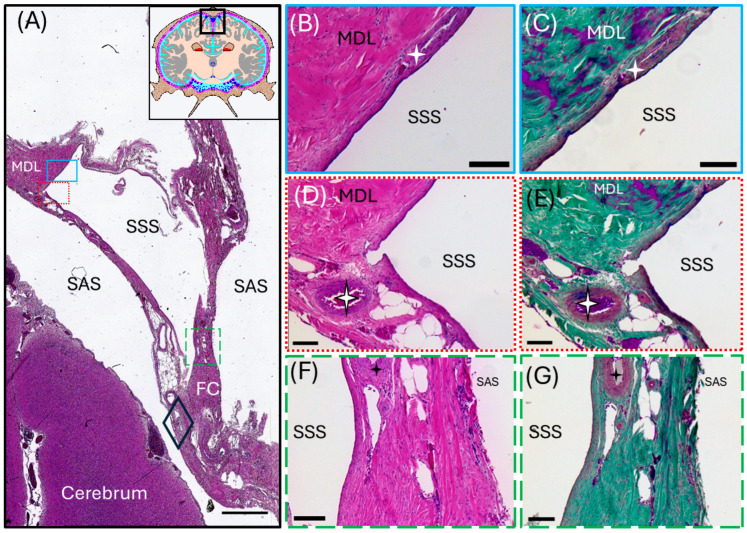
Light micrographs representing a transverse section of the common dolphin (*D. delpis*) superior sagittal sinus displaying the histomorphology of the parasagittal structures, including blood and lymphatic vasculature, with Hematoxylin and Eosin and Masson’s Trichrome. Scale bars represent 100 µm. (**A**–**G**) Animal ID: CAHA559. (**A**) The schematic in the top right corner displays position of the section. A composite photomicrograph of histological section displaying the entire superior sagittal sinus (SSS). Colored boxes correspond to the adjacent images—blue solid box (**B**,**C**), red dotted box (**D**,**E**), and green long-dash box (**F**,**G**). The subarachnoid space (SAS) lies between the meningeal dural layers (MDL) and the cerebrum. The MDLs fuse ventrolaterally around the superior sagittal sinus to form the falx cerebri (FC) which extends ventrally where it separates the two cerebral hemispheres. The periosteal dural layer was lost during dissection. The four-point stars in panels B through G represent three different blood vessels in the surrounding meningeal dural layers surrounding the SSS. The diamond in panel A corresponds to the site of Figure 4 (**A**–**G**).

The superior sagittal sinus and transverse venous sinus are formed between the periosteal and meningeal layers of the dura mater (demonstrated in Figure 1C). The histomorphology of the superior sagittal sinus, which is formed by the separation of the superficial periosteal layer just below the skull and the deeper meningeal dural layers, was revealed using Hematoxylin and Eosin and Masson’s Trichrome (Figure 3). Of note, because of the adherence of the periosteal layer to the skull, this dural layer was partially separated during dissection from the calvarium. The meningeal layers continue deep to the superior sagittal sinus, to form the falx cerebri (FC) (Figure 1B,C and Figure 3A), which descends vertically into the braincase along the longitudinal fissure, dividing the cerebral hemispheres. The superior sagittal sinus of the common dolphin is a large-lumened vessel lined with a thick endothelial layer and basement membrane that was generally lacking a continuous layer of smooth muscle cells and devoid of valves (Figure 3). A thicker layer of smooth muscle cells was observed along the lateral aspects of the sinus, where blood vessels appear to merge with the sinus, suggesting a potential route for cerebral blood drainage. The meningeal dural layers surrounding the superior sagittal sinus comprised thick, fibrous collagen (Figure 3B–E).

### 3.2. Meningeal Lymphatic Vessels

**Figure 4:**
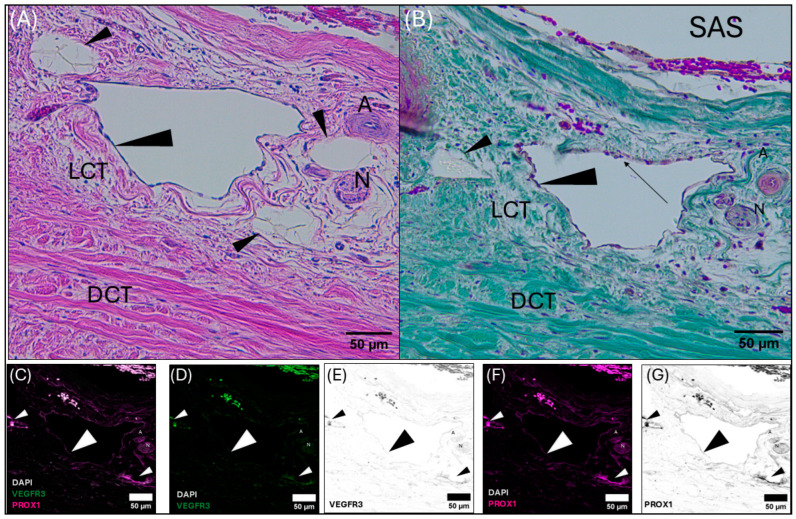
Composite light and confocal micrographs representing a section of the common dolphin (*D. delpis*) meninges adjacent to the superior sagittal sinus (see position at diamond in (**A**)), displaying the histomorphology of the meningeal lymphatic vessels and associated blood vessels within the parasagittal dura mater adjacent to the subarachnoid space (SAS) stained with (**A**) Hematoxylin and Eosin, (**B**) Masson’s Trichrome, and (**C**–**G**) confirmed using immunofluorescent markers for Prox-1 and VEGFR3. Scale bars represent 50 µm. (**A**–**G**) Animal ID: CAHA559. (**A**) A composite photomicrograph of histological section demonstrating a pre-collecting lymphatic vessel (indicated by the large arrowhead), surrounded by initial lymphatics (small arrowheads). Near the collecting lymphatic vessel lies an arteriole (**A**) and a nerve (N). (**B**) Perinuclear bulging of the endothelium of the pre-collecting lymphatic vessel is readily observed, indicated by the black arrow. Initial and pre-collecting meningeal lymphatic vessels, as well as the arteriole and nerve, are seated within loose connective tissue (LCT) surrounded by dense regular connective tissue (DCT). (**C**–**G**) Representative confocal images (individual and merged) from the same section of tissue, demonstrating and differentiating the presence and localization of blood and lymphatic vessels using immunofluorescent markers for vascular endothelial growth factor receptor-3 (VEGFR3) (green) and Prospero homeobox 1 (Prox-1) (magenta), with DAPI (white) counter labeling for nuclei. Note that red blood cells are auto-fluorescent in these images. Merged image (**C**) demonstrates colocalization of Prox-1, VEGFR3, and DAPI. (**D**,**E**) Individually labeled confocal image to illustrate VEGFR3, where black in panel E represents VEGFR3 signal. (**F**,**G**) Individually labeled confocal image to illustrate Prox-1 localization, where black in panel G represents Prox-1 signal.

**Figure 5:**
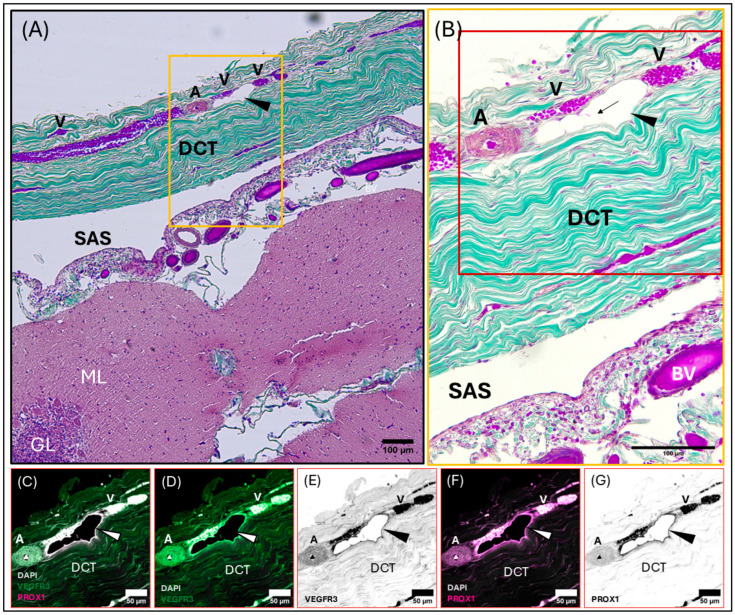
Composite light and confocal micrographs representing a section of the common dolphin (*D. delpis*) meninges adjacent to the transverse venous sinus, displaying the histomorphology of the meningeal lymphatic vessels and blood vessels within the dura mater, stained with (**A**,**B**) Masson’s Trichrome, and (**C**–**G**) confirmed using immunofluorescent markers for Prox-1 and VEGFR3. Scale bars represent 100 µm (**A**,**B**) and 50 µm (**C**–**G**). (**A**–**G**) Animal ID: CAHA559. (**A**) A composite photomicrograph of a histological section demonstrating a pre-collecting lymphatic vessel chain, denoted by the arrowhead, and associated arteriole (A) in cross-section and multiple sections through venules (V) in longitudinal section. Blood vessels and meningeal lymphatic vessels within the dura mater seated within a thick layer of dense regular connective tissue (DCT). Beneath the dura mater lies the subarachnoid space (SAS) and parenchyma of the cerebellum, including the molecular layer (ML) and granular layer (GL) of the gray matter. The yellow box in panel (**A**) denotes the region observed in panel (**B**). (**B**) An intraluminal valve (arrow) can be seen within the bead-like chain of the meningeal lymphatic vessel. The red box in panel (**B**) denotes the region observed using immunofluorescent labeling of lymphatic endothelial cells (**C**–**G**). (**C**–**G**) Representative confocal images (individual and merged) demonstrating and differentiating the presence and localization of blood and lymphatic vessels using immunofluorescent markers for vascular endothelial growth factor receptor-3 (VEGFR3) (green) and Prospero homeobox 1 (Prox-1) (magenta), with DAPI (white) counter labeling for nuclei. Note that red blood cells are auto-fluorescent in these images. Individually labeled confocal images are shown to illustrate VEGFR3 (**D**,**E**) and Prox-1 (**F**,**G**) localization, which can also be observed in the merged image (**C**).

**Figure 6:**
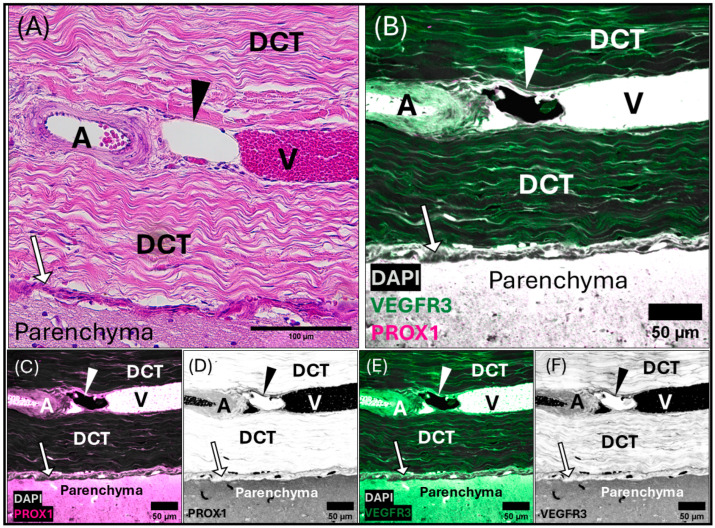
A composite light and confocal micrographs representing a section of the common dolphin (*D. delpis*) meninges adjacent to the transverse venous sinus, displaying the histomorphology of the meningeal lymphatic vessels and associated blood vessels within the dura mater, stained with (**A**) Hematoxylin and Eosin, and (**B**–**F**) confirmed using immunofluorescent markers for Prox-1 and VEGFR3. Scale bars represent 100 µm (**A**) and 50 µm (**B**–**F**). (**A**–**F**) Animal ID: CAHA559. (**A**) A composite photomicrograph of histological section of a singular, sub-circular, initial lymphatic vessel, denoted by the arrowhead, and an associated arteriole (A) in cross-section to the left and venule (V) in longitudinal section to the right, with a shared adventitia. Blood vessels and meningeal lymphatic vessels within the dura mater seated within a thick layer of dense regular connective tissue (DCT). Beneath the dura mater lies the pia mater (arrows) seated above the parenchyma of the cerebellum. (**B**) Merged confocal image demonstrates co-localized labeling of Prox-1 (magenta) and VEGFR3. (**C**,**D**) Strong Prox-1 labeling in the perinuclear region of the lymphatic endothelial cells. (**E**,**F**) Strong VEGFR3 (green) labeling within the cytoplasmic region of the lymphatic endothelial cells. DAPI (white) counter labeling for nuclei. Note that red blood cells are auto-fluorescent in these images. Individually labeled confocal images are shown to illustrate VEGFR3 (**E**,**F**) and Prox-1 (**C**,**D**) localization, which can also be observed in the merged image (**B**).

Meningeal lymphatic vessel identification was achieved by pairing histology (Hematoxylin and Eosin, Masson’s Trichrome) with positive labeling of immunofluorescent antibodies targeting highly conserved proteins (Prospero homeobox protein-1 [Prox-1] and vascular endothelial growth factor receptor 3 [VEGFR3]) found in the endothelial cells of lymphatic vessels. Meningeal lymphatic vessels were identified by the colocalization of Prox-1 and VEGFR3 (Figure 4, Figure 5, Figure 6 and Figures S4–S6) within the endothelial cells lining the lumen of the vessel. Within the lymphatic endothelial cells, the labeling of Prox-1 was seen within the perinuclear region, while VEGFR3 remained within the cytoplasmic region.

Initial meningeal lymphatic vessels are irregularly shaped and consist of a thin-walled, single-cell layer of continuous endothelium (Figure 4). Initial lymphatic vessels feed into larger pre-collecting vessels that gain a basement membrane and discontinuous layer of smooth muscle cells. Eventually, these vessels feed into large collecting vessels characterized by intraluminal valves, a basement membrane, and several layers of smooth muscle cells.

The perinuclear region of the endothelium of meningeal lymphatic vessels is often observed bulging into the lumen (Figure 4B). The endothelial layer of the initial lymphatic vessels surrounding the superior sagittal sinus is backed by loose irregular connective tissue. In some cases, initial lymphatic vessels can be observed on either side of a pre-collecting meningeal lymphatic vessel (Figure 4). In this case, the initial lymphatic vessels present strong Prox-1 and VEGFR3 labeling, whereas the pre-collecting lymphatic vessel presents strong Prox-1 labeling, with weakly positive VEGFR3 labeling (Figure 4).

Meningeal lymphatic vessels confirmed by antibody labeling can be seen in cross (Figure 6, Figures S4A,B,E, S5 and S6A,B,D) and longitudinal sections (Supplementary Figures S4C,D and S6C) and were present throughout the dura of both the superior sagittal sinus and transverse venous sinus. These vessels range in shape from small singular “beads” surrounded by blood vessels (Supplementary Figures S4A,E and S6A), to nets or chains of “beads” (Supplementary Figures S4B and S6D), or large-lumened vessels running along blood vasculature in cross-section (Supplementary Figures S4C,D and S6B,C). Within the meninges of the transverse venous sinus, a pattern of blood vessels (including arterioles and venules) juxtaposed to meningeal lymphatic vessels was frequently observed (Figure 6, Figures S5 and S6B,C). For example, in Supplementary Figure S6B, an arteriole is observed flanked on either side by a thin, collapsed lymphatic vessel in longitudinal section. A similar orientation is seen with a smaller blood vessel, with a larger, thicker-walled collecting lymphatic vessel seen in longitudinal cross-section above (Supplementary Figure S6C). Bead-like, pre-collecting lymphatic vessels can also be seen as a subcircular meningeal lymphatic vessel in cross-section (Figure 6, Figures S5 and S6A) or chains of beads surrounding blood vessels of various sizes (Supplementary Figure S6D).

Micrographs of the transverse venous sinus dura, stained with Masson’s Trichrome, demonstrate the meningeal dura mater and leptomeninges (pia and arachnoid mater) of the cerebellum (Figure 5 and Figure 6). An arteriole can be seen in cross-section juxtaposed to venules (best seen in Figure 5A), sharing an adventitia with a pre-collecting lymphatic vessel. An intraluminal valve is highlighted in Figure 5B. The morphology of the lymphatic vessel highlighted in Figure 5, appearing as a chain of subcircular beads, was observed to be a consistent feature in tissues collected from the transverse sinus. The endothelial layer of initial lymphatics surrounding the transverse venous sinus were commonly embedded within dense regular connective tissue (Figure 5, Figure 6 and Figure S5), as compared to those near the superior sagittal sinus, which were surrounded by loose connective tissue. Meningeal lymphatic vessels often display intraluminal valves, which appear as a V-shape in cross-section (Supplementary Figure S6A) or chain of beads in longitudinal section (Figure 5, Figure 6 and Figure S6D).

### 3.3. Perivascular Spaces and AQP4 Expression

**Figure 7:**
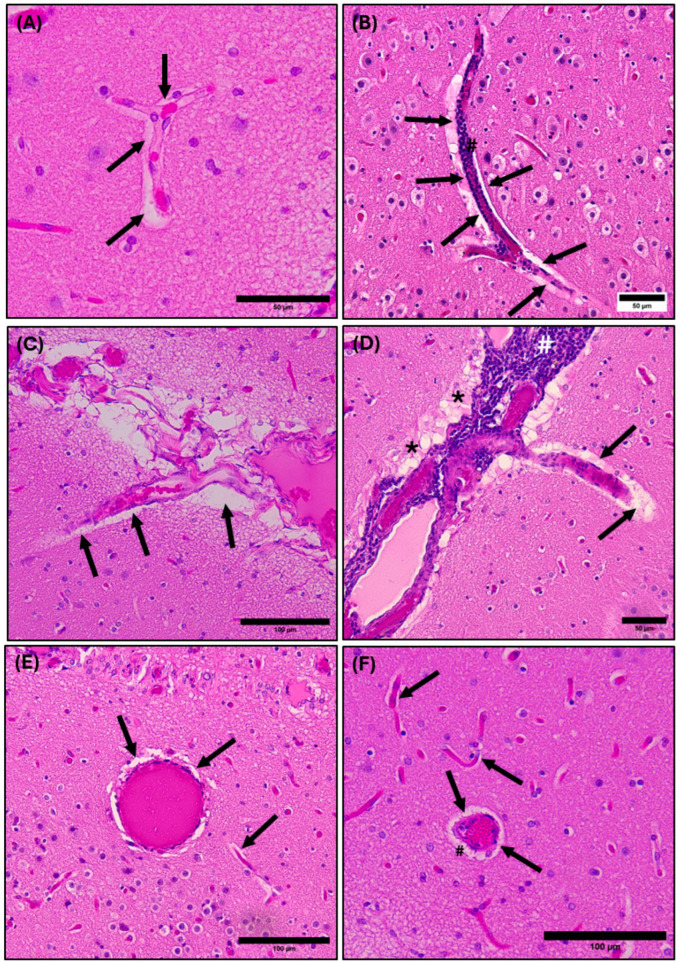
Photomicrographs of the common dolphin (*D. delpis*) cerebral parenchyma, stained with Hematoxylin and Eosin, displaying the presence and morphology of perivascular spaces. Scale bars represent 50 µm (**A**,**B**,**D**) and 100 µm (**C**,**E**,**F**). (**A**,**C**,**F**) Animal ID: CAHA559; (**B**,**D**,**E**) Animal ID: JPIER040. (**A**) A light micrograph demonstrating the normal morphology of the perivascular space (arrows) surrounding a branching blood vessel within the cerebrum. (**B**) A light micrograph demonstrating perivascular cuffing of leukocytes (#) within the perivascular space (arrows) of a branching blood vessel. (**C**) A light micrograph demonstrating the normal morphology of the perivascular space (arrows) surrounding leptomeningeal blood vessels diving into the brain parenchyma. (**D**) A light micrograph demonstrating the pathological morphology of lymphocytic infiltration and perivascular cuffing (#) within the perivascular space (arrows) of leptomeningeal blood vessels diving into the brain parenchyma. Additionally, edemic tissue is present (*). (**E**) A light micrograph of normal morphology of the perivascular space (arrows) in cross- and longitudinal section within the brain parenchyma. (**F**) A light micrograph of morphology of the perivascular space (arrows) with minor perivascular cuffing (#) see in cross-section.

**Figure 8:**
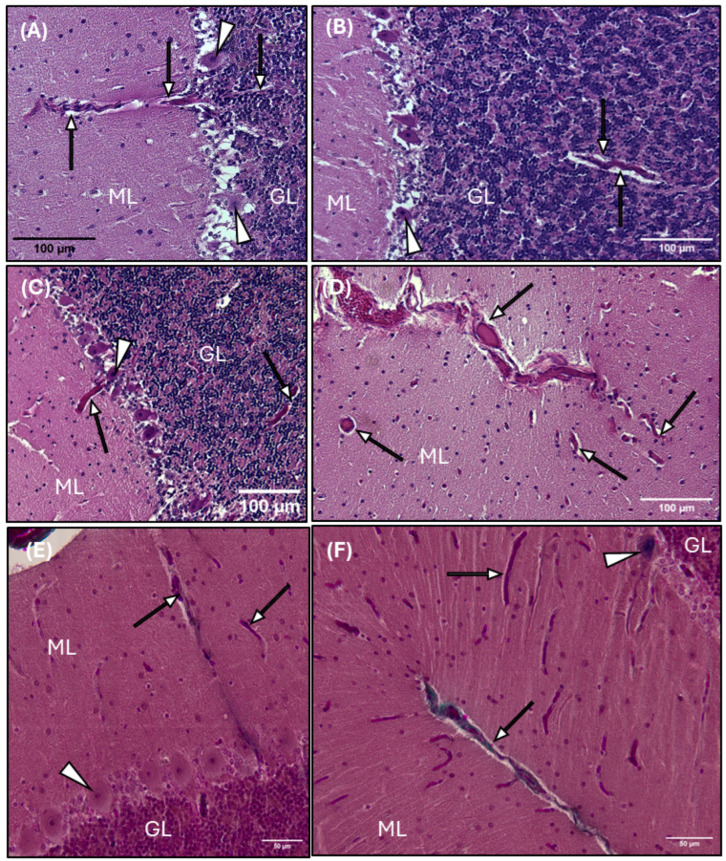
Photomicrographs of the common dolphin (*D. delpis*) cerebellar parenchyma, stained with Hematoxylin and Eosin (**A**–**D**) and Masson’s Trichrome (**E**,**F**), displaying the presence and morphology of perivascular spaces within the gray matter and granular layer. Scale bars represent 100 µm (**A**–**D**) and 50 µm (**E**,**F**). (**A**–**F**) Animal ID: CAHA559. (**A**–**C**,**E**) A light micrograph demonstrating the morphology of the perivascular space (arrows) within the molecular layer (ML) and granular layer (GL), closely associated with highly metabolic Purkinje neurons (arrowheads). (**D**,**F**) Perivascular space morphology (arrows) along blood vasculature diving into the cerebellum sulcus, as well as perivascular space (arrows) surrounding blood vessels in cross-section (**D**) and longitudinal/oblique section (**F**).

The perivascular space follows penetrating blood vessels from the subarachnoid space deep into the brain tissue, providing a pathway for fluid to flow into and out of the brain. A distinct halo is observed surrounding blood vessels within the gray and white matter of both the cerebrum (Figure 7) and cerebellum (Figure 8), demonstrating the presence and morphology of perivascular compartments surrounding blood vessels in the common dolphin.

Perivascular spaces surrounding blood vessels in the cerebral parenchyma of the common dolphin demonstrated both normal morphology (Figure 7A,C,E) and pathology (Figure 7B,D,F). In some cases, inflammation from infiltration of leukocytes along the outer walls of the blood vessels demonstrated acute perivascular cuffing. Perivascular cuffing is defined as infiltration and accumulation of leukocytes, lymphoid cells, and macrophages within the perivascular space between the endothelial and parenchymal membranes [73,74,75]. Perivascular cuffing was observed along the leptomeninges of blood vasculature diving into the cerebrum (Figure 7B,D) and in cross-section of a cerebral blood vessel (Figure 7F).

A large cerebral branching blood vessel can be seen with a distinct white halo representing the perivascular space (Figure 7C,D). There is also infiltration of leukocytes along the outer vascular wall, causing perivascular cuffing and brain tissue surrounding the blood vessel shows evidence of cerebral vascular edema (Figure 7D). The dysregulation of glymphatic function leads to a lack of integrity of the fluid barrier, leading to edema, or swelling of the brain tissue, causing it to appear sponge-like. The edema is noticeable surrounding the leptomeninges in Figure 7D, where the tissue is lighter in appearance and takes on a sponge-like texture.

Blood vessel histomorphology and the associated annular perivascular space are observed throughout the cerebellum, including the gray matter, white matter, and granular layer (Figure 8). A distinct white halo is observed surrounding blood vessels diving deep into the vascular cul-de-sac, or the invagination of the sulcus, of the cerebellum (Figure 8D–F), penetrating the gray matter (Figure 8A,C–F) and around the highly metabolic Purkinje cells (Figure 8C), and through the granular layer (Figure 8F).

**Figure 9:**
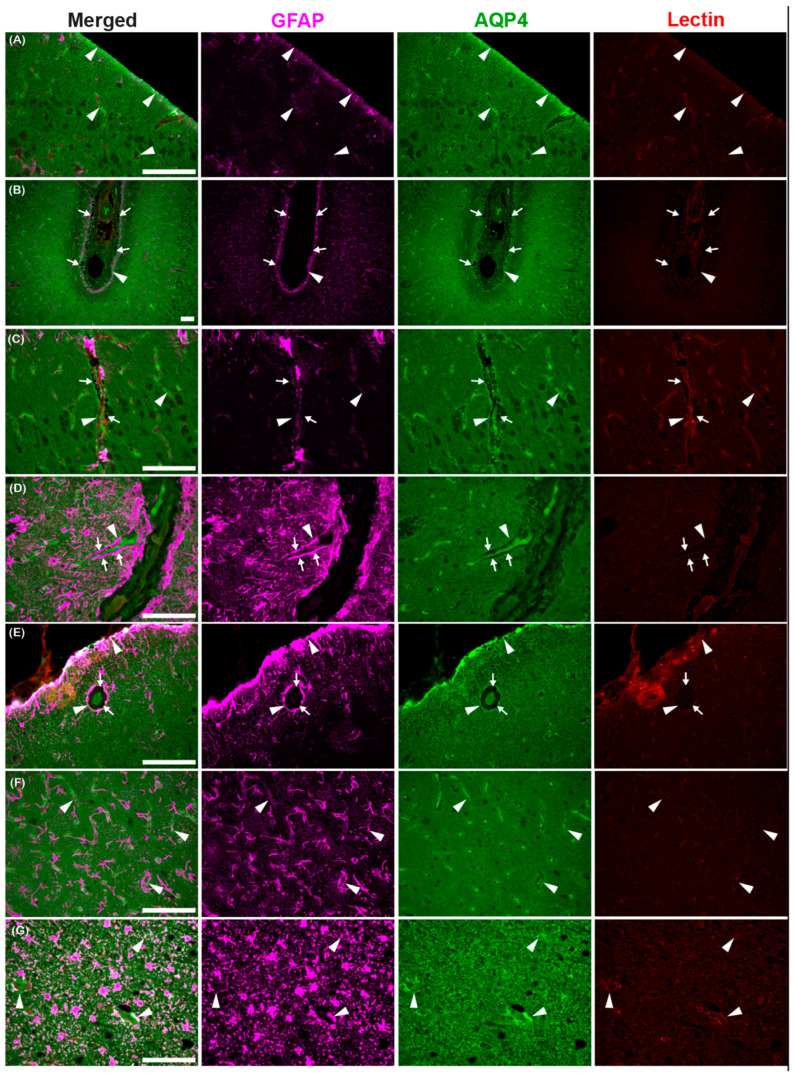
Photomicrographs of glial fibrillary acidic protein (GFAP), aquaporin-4 (AQP4), and lectin immunoreactivity in the cerebral cortex of the common dolphin (*D. delpis*). Scale bars represent 100 µm (**A**–**G**). Animal IDs: (**A**) CAHA559, (**B**) JPIER40, (**C**) CAHA560, (**D**) JPIER40, (**E**) CAHA559, (**F**) JPIER40, and (**G**) CAHA559. Representative full focus z-stack images of widespread expression of AQP4 (green) on astrocytes (GFAP, magenta) surrounding blood vessels (lectin, red) throughout the cortex. (**A**) Representative image of the cerebral cortical surface with AQP4 enhancement (arrowheads) at the glia limitans. (**B**) Sulcus with AQP4 enhancement (arrowheads) at the glia limitans and perivascular spaces (arrows) surrounding pial vessels. (**C**–**E**) Perivascular spaces (arrows) surrounding penetrating vessels and AQP4 enhancement (arrowheads) surrounding large vessels and capillaries. (**F**,**G**) AQP4 surrounding capillaries in the (**F**) cortical gray matter and (**G**) subcortical white matter.

**Figure 10:**
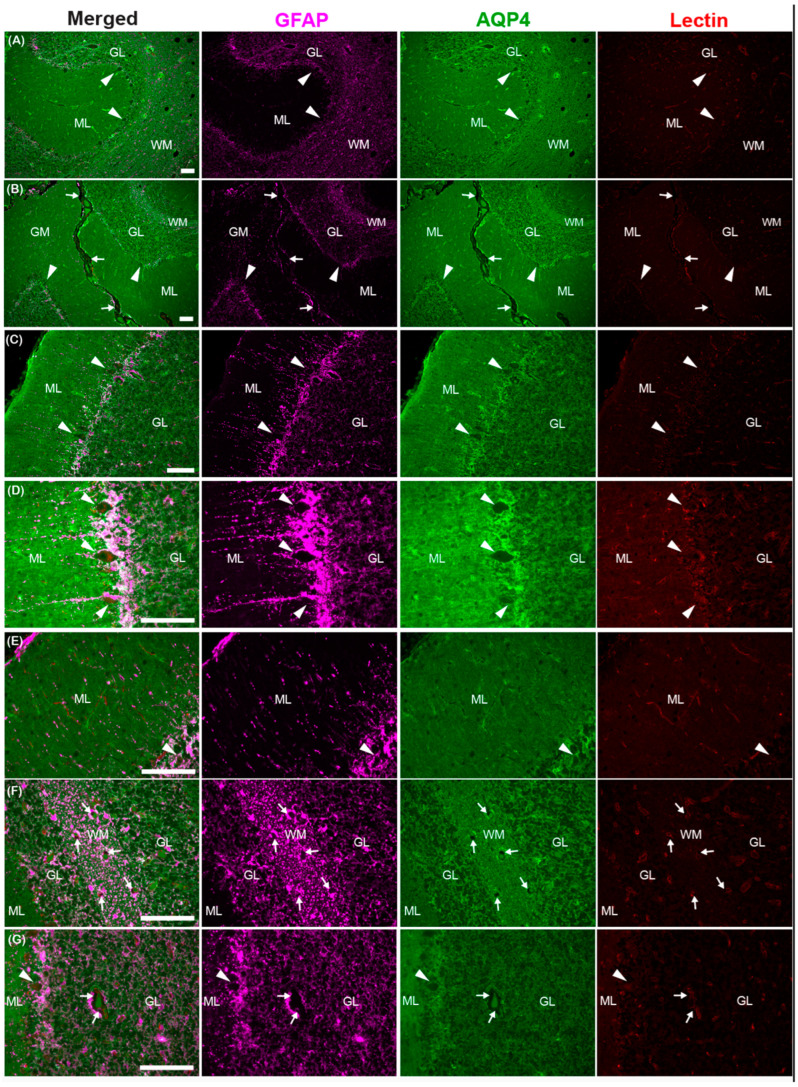
Photomicrographs of glial fibrillary acidic protein (GFAP), aquaporin-4 (AQP4), and lectin immunoreactivity in the cerebellum of the common dolphin (*D. delpis*). Scale bars represent 100 µm (**A**–**G**). Animal IDs: (**A**) CAHA560, (**B**) CAHA559, (**C**–**E**) JPIER40, (**F**) CAHA559, and (**G**) JPIER40. Representative full focus z-stack images of wide-spread expression of AQP4 (green) on astrocytes (GFAP, magenta) surrounding blood vessels (lectin, red) throughout the cerebellum. (**A**,**B**) Representative images of cerebellar sulci showing widespread AQP4 (green) on astrocytes (magenta) throughout the molecular layer (ML), Purkinje cell (arrowheads) layer, granular layer (GL), and white matter (WM). (**C**,**D**) High expression of AQP4 and GFAP surrounding highly metabolic Purkinje cells (arrowheads). (**E**) Representative image of perivascular enrichment of AQP4 surrounding capillaries in the molecular layer (ML). (**F**,**G**) Perivascular spaces surrounding large vessels in the white matter (**F**) and granular layer (**G**).

The astroglial water channel AQP4, which facilitates glymphatic exchange, is expressed on the endfeet of astrocytes lining perivascular spaces throughout the brains of the common dolphin (Figure 9 and Figure 10). AQP4 and the astrocyte marker glial-fibrillary acidic protein (GFAP) were enriched at the glia limitans and surrounding microvasculature throughout the cortex (Figure 9) and cerebellum (Figure 10), similar to what has been reported in bottlenose dolphin [21] and the human brain [76,77]. Enrichment of AQP4 and high GFAP positivity were also observed in the Purkinje cell layer unique to the cerebellum (Figure 10). Individual GFAP-positive cells tended to be more visible in the gray matter while more mesh-like networks were observed in the white matter, as previously reported [78].

## 4. Discussion

This study demonstrates that the common dolphin (*D. delphis*)—an offshore delphinid—possesses all structural components required for functional glymphatic and meningeal lymphatic systems, including the glial-dependent network of perivascular spaces and AQP4 water channels surrounding blood vessels within the parenchyma, meningeal lymphatic vessels within the surrounding dural layers, and cranial dural venous sinuses draining cerebral blood. Thus, common dolphins, like bottlenose dolphins (*T. truncatus*), possess the anatomical structures necessary to distribute CSF deep into the brain tissue, clear waste from within the parenchyma, and modulate fluid dynamics within the brain.

The superior sagittal sinus is a valveless vein seated along the midline of the cerebral hemispheres, which is an important drainage site for cerebral and cerebellar veins. Additionally, the superior sagittal sinus is an important site for the collection and drainage of CSF through arachnoid granulations that perforate the sinus.

In terrestrial mammals, this sinus has been divided into three regions with distinct characteristics—the frontal, parietal, and occipital superior sagittal sinuses [79,80,81]. In humans, the superior sagittal sinus begins as a simple, longitudinal channel rostrally but becomes increasingly complex caudally, developing a network of bridging vessels that traverse the subdural space and connect to the confluence of the transverse sinus. Disruption of these bridging vessels is one of the leading causes of acute subdural hematoma in humans [81]. Additionally, there is evidence of differences in the structural composition of the superior sagittal sinus along its caudal progression. In the common dolphin, the position and trajectory of the superior sagittal sinus within the cranial vault and its connections to the transverse sinus, as confirmed by CT angiography, are consistent with that of terrestrial mammals [79,80,81], apart from the more elaborate network of vascular structures exiting the skull via the ethmoid plate. Additionally, the gross histomorphology of the superior sagittal sinus was consistent with descriptions in terrestrial mammals, within the regions investigated in the common dolphin.

In rodents, lymphatic vessels lie alongside blood vessels in the meninges and surround the dural venous sinuses [11,82,83,84,85]. The network of lymphatic vessels surrounding the rodent transverse venous sinus has been reported to be larger and more complex than those surrounding the superior sagittal sinus which resemble initial lymphatics that extend throughout the entire dural layer [85]. Collecting lymphatic vessels are also observed at the base of the skull and extend along the internal jugular vein where they leave the skull reviewed by [11,82,83,86]. In the common dolphin, meningeal lymphatic vessels were found throughout the dura surrounding the superior sagittal and transverse venous sinuses. Vessels near the superior sagittal sinus were closely associated with arterial vessels and nerves and surrounded by loose connective tissue backed by dense regular connective tissue. Within the transverse sinus, the meningeal lymphatic vessels observed were commonly part of a vascular triad, tightly grouped with arterial and venous vessels within dense dural connective tissue. Thus, the common dolphin possesses meningeal lymphatic vessels within the connective tissues of their dural sinus, consistent with terrestrial mammal studies to date [13,83,86,87,88] and similar to the recently investigated bottlenose dolphin [21].

The morphology of perivascular spaces in the common dolphin was also consistent with those seen in terrestrial mammals, including humans, non-human primates, and rodents [18,19,20], as well as those of the bottlenose dolphin [21]. We observed widespread expression of AQP4 throughout the cerebrum and cerebellum, in both white and gray matter, as well as enrichment of AQP4 surrounding vessels and at the glia limitans, consistent with what has been reported more extensively in terrestrial mammals. AQP4 expression was shown in relation to the astrocyte cell marker GFAP. We observed widespread expression of the astroglial marker GFAP throughout the brain and surrounding blood vessels, similar to the glial cell architecture that has been previously reported in delphinids [33,89]. Similar to AQP4, GFAP expression was high at the glia limitans, and GFAP-positive endfeet were observed surrounding both large vessels and capillaries. The characterization of these perivascular spaces, lined by AQP4 and GFAP, provides evidence that the common dolphin brain possesses the anatomical requirements for a functional glymphatic system.

A greater understanding of the brain’s glymphatic and meningeal lymphatic systems within diving mammals can provide a foundation for comparative research studies for both marine mammal and human health. The delphinid central nervous system anatomy is a comparative, natural model for humans, given their long life spans, large relative brain mass (encephalization), and significant gyrification of the neocortex [90,91]. Marine mammals offer an opportunity to investigate human neuropathies in apnea-adapted species that routinely experience alterations to the physiological drivers of the glymphatic and meningeal lymphatic systems. For example, despite their diving capabilities, there is evidence they are vulnerable to hypoxic insult and CNS neuropathologies, such as Alzheimer’s disease and decompression illness [51,52,53,54,55,56,57,58], highlighting their importance as a comparative model. Thus, studying delphinid central nervous system (CNS) anatomy, specifically within the contexts of the brain glymphatic and meningeal lymphatic systems, may offer valuable insights into both diving physiology and mammalian brain health.

As an example, damage to the dural venous sinuses in terrestrial mammals, including humans, can alter intracranial blood pressures, cause hemorrhage, and can result in death [80,81,92,93,94,95]. Acute occlusion of the superior sagittal sinus through injury (i.e., acoustic, blast, concussive), pathology (meningioma), or surgical intervention can significantly increase intracranial pressure and cerebral blood volume [80,96,97]. Several pelagic delphinids are known to be susceptible to CNS injuries linked to mid-frequency active sonar (MFAS), underwater blasts [60,61,98,99], acute trauma from vessel strike, and headbutting behaviors [90]. Mass-stranding events associated with naval sonar or underwater blasts can result in gas emboli lesions due to alteration of their diving behavior or CNS hemorrhages [54,60,61,99,100]. Furthermore, live-stranding events are marked by progressive cardiovascular and pulmonary insults, leading to vascular perfusion changes and hypertension, otherwise known as “stranding stress” [100]. Given the critical role of the dural venous sinuses in CNS health, understanding their structure is fundamental for the development of medical interventions and treatments [5]. The results of our work demonstrate that the common dolphin possesses the structural components required for a functioning glympjhatic and meningeal lymphatic system. However, it would be valuable to explore how intracerebral and intracranial fluid dynamics are altered by diving physiology, which would require functional imaging studies.

Impairment or dysfunction of glymphatic or meningeal lymphatic function can lead to neurological disorders due to the accumulation of toxic proteins [101]. Diseases that affect the central nervous system, such as vascular disorders, neurodegenerative diseases, inflammatory diseases, and traumatic brain injury, are all marked by a disruption in the homeostatic functions regulating waste disposal and immune cell regulation [102]. This study documented several pathologies, including leukocyte infiltration and edematous tissue within the perivascular spaces surrounding blood vessels within the brain. Similarly, lymphocytic infiltration of the meninges is also a commonly observed pathology in the common dolphin from the waters of Spain, due to viral and bacterial infections [103,104,105], highlighting the importance of further investigating the role of the glymphatic and meningeal lymphatic systems in this species across disease states.

It is important to acknowledge the limitations of this study and the logistical challenges that exist when working with protected marine mammal species [15]. Most in vivo neuroanatomical studies on marine mammals are not possible due to ethical concerns and legal protections. Additionally, significant logistical challenges exist when working with marine mammal species, including skull and brain size, access to fresh tissue, and the stochastic nature of stranding events. Studies utilizing stranded marine mammal tissues cannot control for life history variables such as sex, age, or disease status. However, despite these limitations, stranded tissues provide valuable insight into naturally occurring pathologies, paralleling postmortem studies in humans.

Dolphins act as key indicators of coastal ecosystem health due to their longevity, their consistent presence in coastal marine habitats, and their tendency to accumulate contaminants through bioaccumulation [106]. The examination of stranded animals provides valuable insights into the health of marine mammal populations and the coastal marine ecosystems they inhabit, informing us of risks to both animal and human CNS health. Considering their vulnerabilities to marine pollutants and high degree of cortical gyrification, delphinid species provide a valuable comparative natural model for comprehending analogous neuropathologies found in humans and examining how the brain changes in a demanding environment [107]. Future directions should consider the link between the glymphatic and meningeal lymphatic systems and the development of neurodegenerative pathologies linked to environmental toxins and pollutants, such as mercury [108] and per- and polyfluorinated substances (PFAS) [109], fine particulate matter exposure [110], and cyanobacterial toxins [62].

## 5. Conclusions

This study demonstrates that the common dolphin (*D. delphis*), a pelagic delphinid, possesses the anatomical structures of the glymphatic and meningeal lymphatic systems including the dural venous sinuses, meningeal lymphatic vessels, perivascular spaces, and astroglial AQP4 water channels. Thus, like the terrestrial mammals studied to date, and the shallow-diving coastal bottlenose dolphin, the common dolphin displays the structural components necessary for functional glymphatic and meningeal lymphatic systems. Our results provide novel insights into the structural components of these fluid and waste-maintenance systems in a diving marine mammal. This study also demonstrated pathologies of the perivascular system, including inflammation, leukocyte infiltration and edema. This work provides a foundation for further comparative investigations into the function of these systems in other marine mammal species and may offer insights into both diving physiology and mammalian brain health.

## Figures and Tables

**Table 1:** Common dolphin (*D. delphis*) specimens collected and utilized in this study. Due to the opportunistic nature of stranding events this study could not control for sex or life history categories in these specimens. The asterisk on IFAW12-364 denotes the animal was used solely for computed tomography investigation.

Animal ID	Species	Strand Date	SI Code	Sex	Life History Category	Total Length (cm)
IFAW-12-364 *	*Delphinus delphis*	25-Nov-2012	1	Female	Subadult	210
CAHA559	*Delphinus delphis*	15-Feb-2022	1	Male	Subadult	191
CAHA560	*Delphinus delphis*	15-Feb-2022	1	Male	Subadult	179
JPIER040	*Delphinus delphis*	26-Feb-2023	2	Male	Subadult	202.5

**Table 2:** Antibodies used to identify lymphatic endothelial cells in the common dolphin (*D. delpis*). Primary and secondary antibody information for Prospero homeobox-1 protein (Prox-1) and vascular endothelial growth factor-3 (VEGFR3) (1:200 dilution).

Antigen	Antibody Species/ Isotype	Target Species	Class	Suppliers	Concentration
VEGFR3	Goat IgG	Human	Polyclonal	R&D Inc., Minneapolis, MN, USA (PN:AF349)	0.2 µg/mL
Prox-1	Rabbit IgG	Human, mouse, rat, zebrafish	Polyclonal	AngioBio Inc., San Diego, CA, USA (PN:11-002P)	10 µg/mL
**Secondary Antibody**					
Cy-5-conjugated donkey anti-goat IgG				Jackson Immunoresearch (705-175-147)	2.5 µg/mL
Cy-3-conjugated donkey anti-rabbit IgG				Jackson Immunoresearch (711-165-152)	2.5 µg/mL

**Table 3:** Percentage of shared identity of common lymphatic endothelial cell protein markers between human (*Homo sapiens*) and the common dolphin (*D. delpis*). A protein sequence alignment using NCBI BLAST was conducted to identify highly conserved genes and epitopes. From this analysis, we selected Prox-1 and VEGFR3 proteins for the antibody labeling of meningeal lymphatic vessels.

Protein	Percentage of Shared Identity to Human Protein
LYVE-1	74.84%
PROX-1	99.32%
VEGFR3	88.08%
Podoplanin	50.20%

## Data Availability

All data are available within the text and Supplemental Materials.

## Supplementary Materials

The following supporting information can be downloaded at: https://www.mdpi.com/article/10.3390/ani15050729/s1. Figure S1: Light micrograph representing a section of the common dolphin (*Delphinus delphis*) small intestine stained with Hematoxylin and Eosin displaying the histomorphology of lymphatic vessels; Figure S2: Confocal micrograph representing a section of the common dolphin (*Delphinus delphis*) small intestine confirming functionality of immunofluorescent markers; Figure S3: Confocal micrograph representing a section of the common dolphin (*Delphinus delphis*) meninges to confirm functionality of immunofluorescent markers; Figure S4: Confocal photomicrographs of representative sections of the common dolphin (*Delphinus delphis*) from the parasagittal meninges surrounding the superior sagittal sinus (SSS) using immunofluorescent markers; Figure S5: Composite light and confocal micrographs representing a section of the common dolphin (*Delphinus delphis*) meninges adjacent to the transverse venous sinus, displaying the histomorphology of the meningeal lymphatic vessels and associated blood vessels within the dura mater, stained with (A) Hematoxylin and Eosin, and (B–F) confirmed using immunofluorescent markers for Prox-1 and VEGFR3; Figure S6: Confocal photomicrographs of representative sections of the common dolphin (*Delphinus delphis*) from the para-sagittal meninges surrounding the transverse venous sinus (TVS) using immunofluorescent markers; Table S1: Computed tomography scanning parameters for the common dolphin (*Delphinus delphis*) cranial venous vasculature examination.
