# Supplementary material for: Meningeal Lymphatic and Glymphatic Structures in a Pelagic Delphinid (Delphinus delphis)
Source: Animals (Basel). 2025 Mar 4;15(5):729. doi: 10.3390/ani15050729 (PMC11899484; doi:10.3390/ani15050729)
Supplement: Supplementary file 1 [file animals-15-00729-s001.zip › animals-3482169-supplementary.pdf]

Supplemental Materials

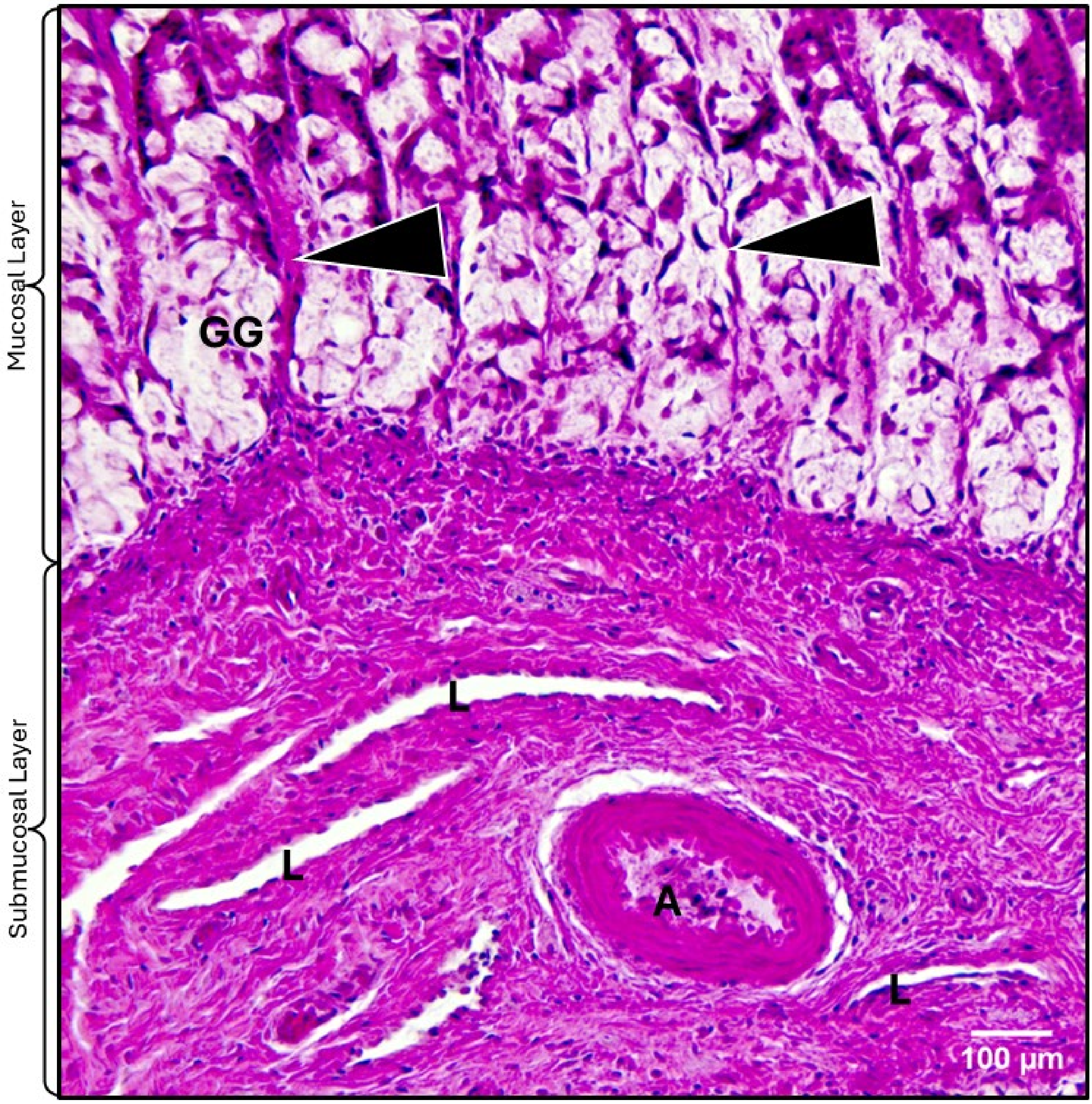

Figure S1. Light micrograph representing a section of the common dolphin (*Delphinus delphis*) small intestine stained with Hematoxylin and Eosin displaying the histomorphology of lymphatic vessels. Scale bar represents 100 µm. Animal ID: CAHA560. Several lymphatic vessels (L) in longitudinal section within the submucosa. A = artery. Lymphatic vessels extend up (arrowheads) into the mucosal layer between and parallel to columns of gastric glands (GG).

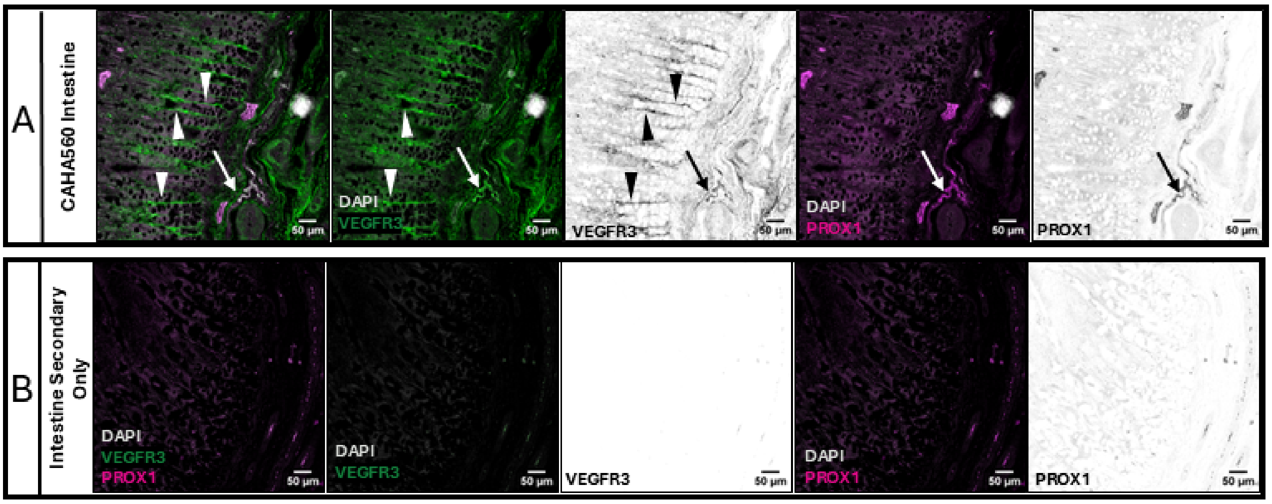

Figure S2. Confocal micrograph representing a section of the common dolphin (Delphinus delphis) small intestine confirming functionality of immunofluorescent markers. Scale bar represents 50 µm. (A-B) Animal ID: CAHA560. Representative confocal photomicrographs demonstrating the presence and localization of blood and lymphatic vessels from the small intestine of the common dolphin displayed with merged and individual immunofluorescent labeling using vascular endothelial growth factor receptor-3 (VEGFR3) (green) and Prospero homeobox 1 (Prox-1) (magenta), with DAPI (white) counter labeling for nuclei. Note that red blood cells present are auto-fluorescent in these images. (A) Common dolphin intestinal tissue labeled with primary and secondary antibodies for Prox-1 and VEGFR3. Morphologically identified meningeal lymphatic vessel (arrows) sits within the submucosa with strong VEGFR3 and Prox-1 signal observed. Lacteals labeled strongly with VEGFR3 (arrowheads). (B) Common dolphin intestinal tissue labeled with Prox-1 and VEGFR3 secondary antibodies only. Absent to weak VEGFR3 and Prox-1 signal is observed.

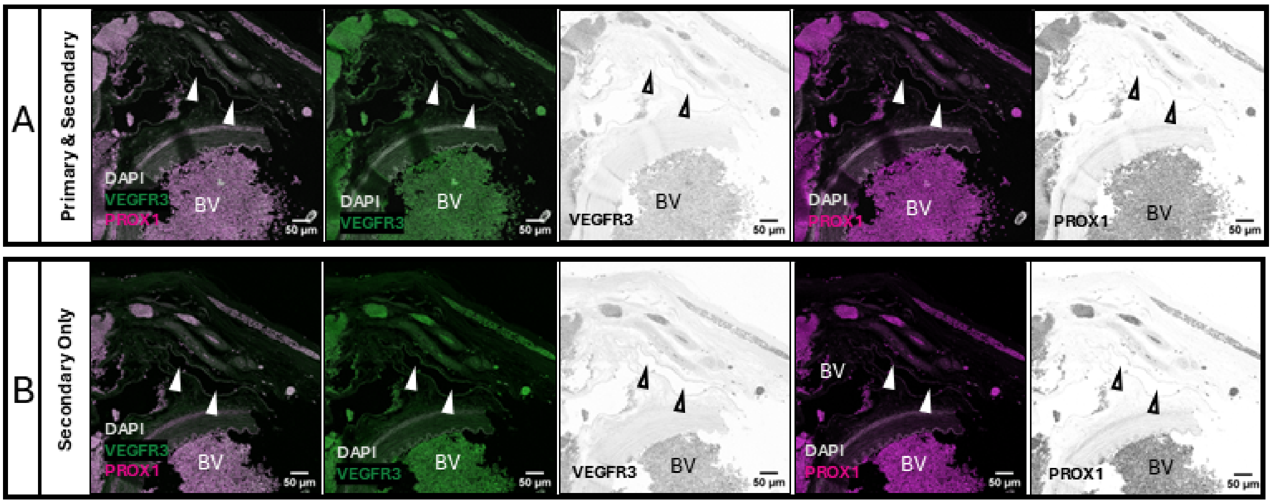

Figure S3. **Confocal micrograph representing a section of the common dolphin (*Delphinus delphis*) meninges to confirm functionality of immunofluorescent markers. Scale bar represents 50 µm.** (A-B) Animal ID: JPIER040. To confirm the functionality of immunofluorescent markers, used to identify lymphatic endothelial cells, tissue from the same region of common dolphin meningeal tissue was labeled. Representative confocal images (individual and merged) demonstrating and differentiating the presence and localization of blood and lymphatic vessels using immunofluorescent markers for vascular endothelial growth factor receptor-3 (VEGFR3) (green) and Prospero homeobox 1 (Prox-1) (magenta), with DAPI (white) counter labeling for nuclei. Note that red blood

cells are auto-fluorescent in these images. (A) Common dolphin meningeal tissue labeled with primary and secondary antibodies for Prox-1 and VEGFR3. Morphologically identified meningeal lymphatic vessel (arrowheads) sits within the dura above a blood vessel (BV). A strong VEGFR3 and moderate Prox-1 signal are observed. (B) Common dolphin meningeal tissue labeled with Prox-1 and VEGFR3 secondary antibodies only. Morphologically identified meningeal lymphatic vessel (arrowheads) sits within the dura above a blood vessel (BV).

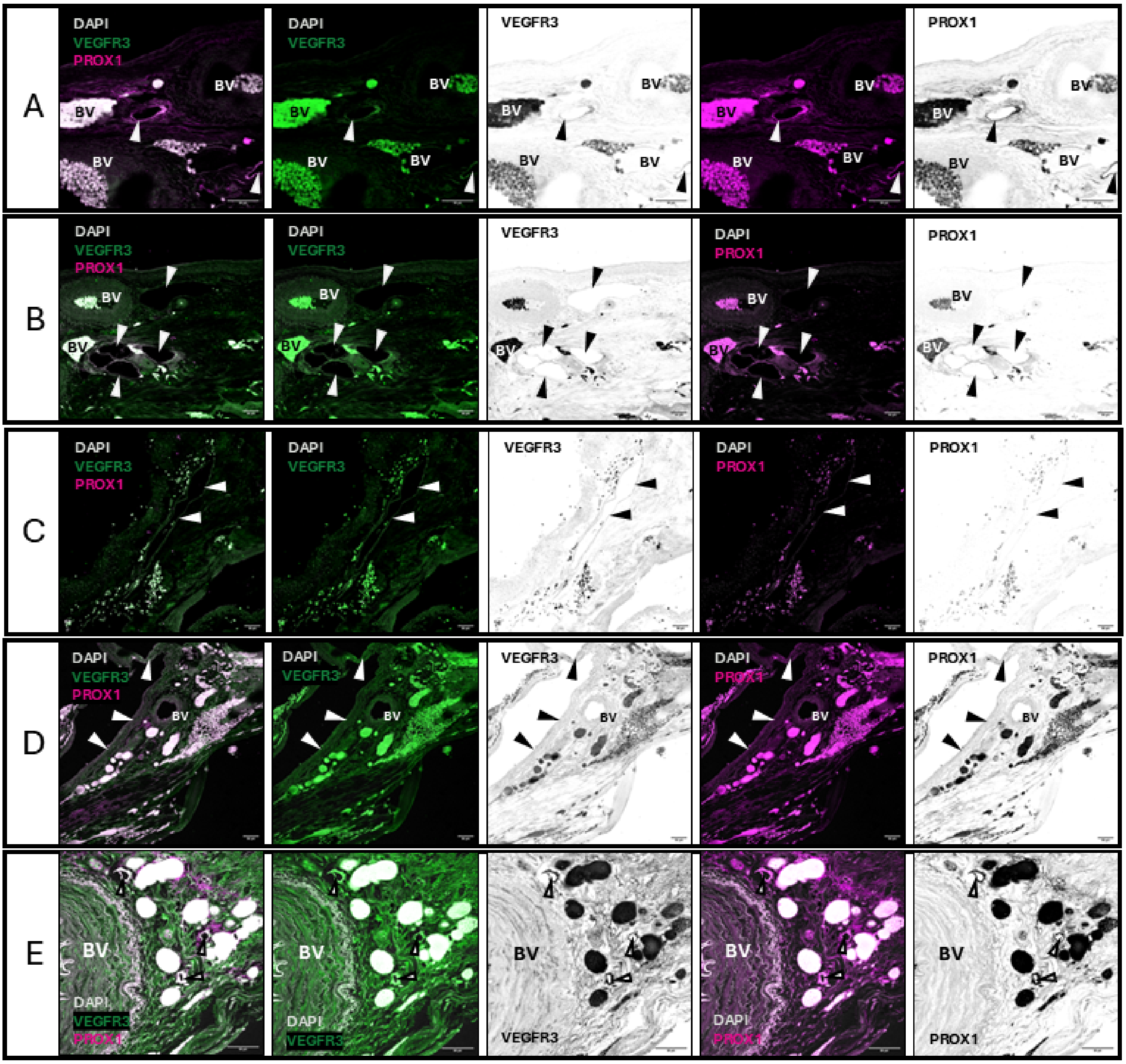

Figure S4. Confocal photomicrographs of representative sections of the common dolphin (*Delphinus delphis)* from the parasagittal meninges surrounding the superior sagittal sinus (SSS) using immunofluorescent markers. Scale bars represent 50 μm. (A – C) Animal ID: CAHA559; (D-E) Animal ID: JPIER040. (A-E) Confocal images of the superior sagittal sinus displaying merged and individual fluorescent markers for vascular endothelial growth factor receptor-3 (VEGFR3) (green) and Prospero homeobox 1 (Prox-

1) (magenta), with DAPI (white) counter labeling for nuclei. (A-B, E) Meningeal lymphatic vessels (arrowheads) in cross-section with strong Prox-1 positive labeling of lymphatic endothelial cells. VEGFR3 displays strong colocalized positive labeling with Prox-1. (C-D) Meningeal lymphatic vessels (arrowheads) in longitudinal-section with strong Prox-1 positive labeling of lymphatic endothelial cells with strong colocalization with labeling of VEGFR3. (A-E) A consistently noted feature is the association of meningeal lymphatic vessels are surrounded by several blood vessels (BV). Note that red blood cells are auto-fluorescent in these images.

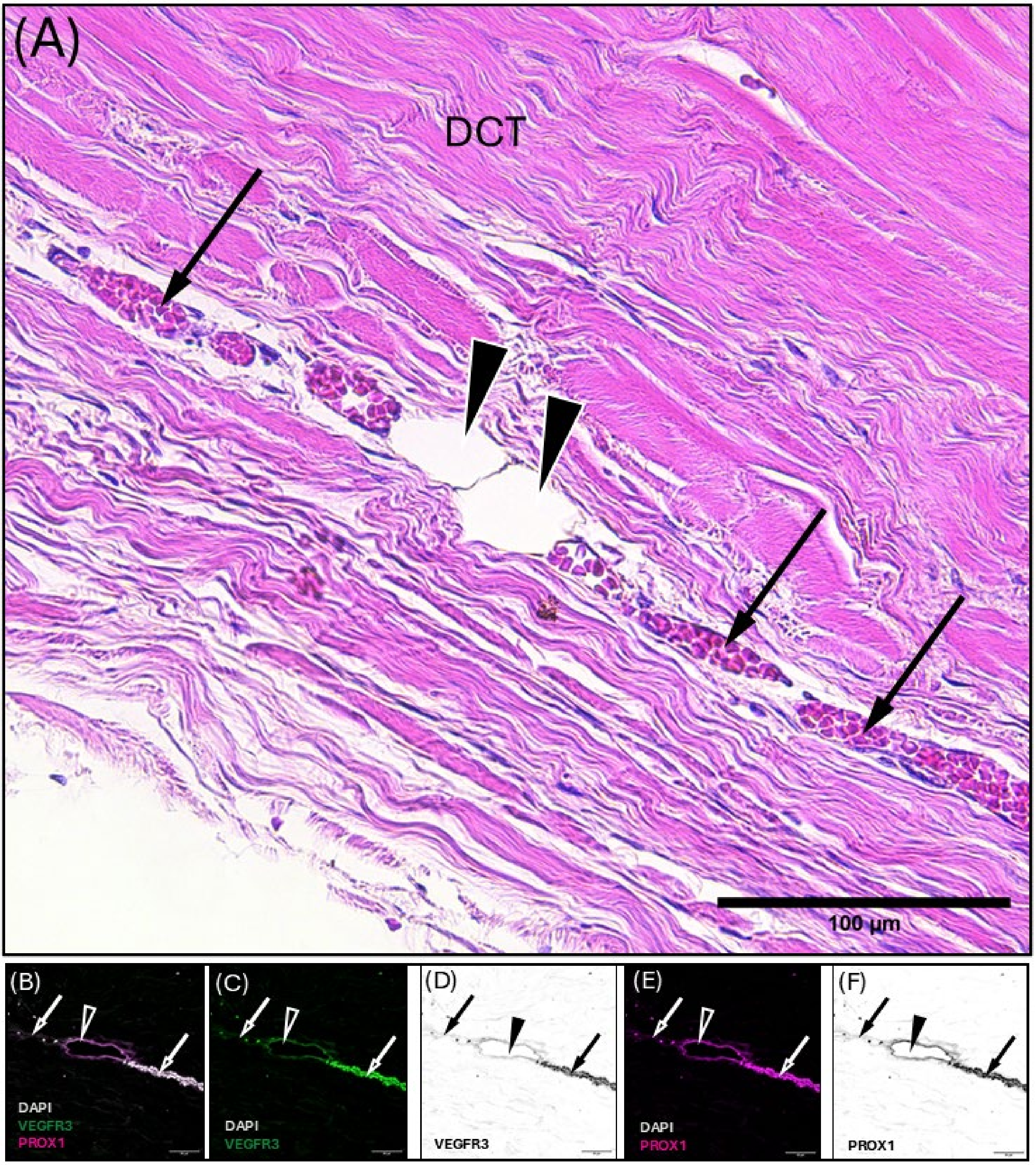

Figure S5. Composite light and confocal micrographs representing a section of the common dolphin (*Delphinus delphis*) meninges adjacent to the transverse venous sinus, displaying the histomorphology of the meningeal lymphatic vessels and associated blood vessels within the dura mater, stained with (A) Hematoxylin and Eosin, and (B-F) confirmed using immunofluorescent markers for Prox-1 and VEGFR3. Scale bars represent 100 µm (A) and 50 µm (B-F). (A-F) Animal ID: CAHA559. (A) A composite photomicrograph of histological section demonstrating the structural arrangement of a two bead-like segments of initial meningeal lymphatic vessels, denoted by the arrowheads, surrounded by venules in longitudinal section (arrows) on either side. (B) Merged confocal

image demonstrates co-localized labeling of Prox-1 and VEGFR3. (C-D) Moderate VEGFR3 labeling in the cytoplasmic region of the lymphatic endothelial cells. (E-F) Strong Prox-1 labeling within the perinuclear region of the lymphatic endothelial cells. Representative confocal images (individual and merged) demonstrating and differentiating the presence and localization of blood and lymphatic vessels using immunofluorescent markers for vascular endothelial growth factor receptor-3 (VEGFR3) (green) and Prospero homeobox 1 (Prox-1) (magenta), with DAPI (white) counter labeling for nuclei. Note that red blood cells are auto-fluorescent in these images. Individually labeled confocal images are shown to illustrate VEGFR3 (C, D) and Prox-1 (E, F) localization, which can also be observed in the merged image (B). Blood vasculature and meningeal lymphatic vessels within the dura mater seated within a thick layer of dense regular connective tissue (DCT).

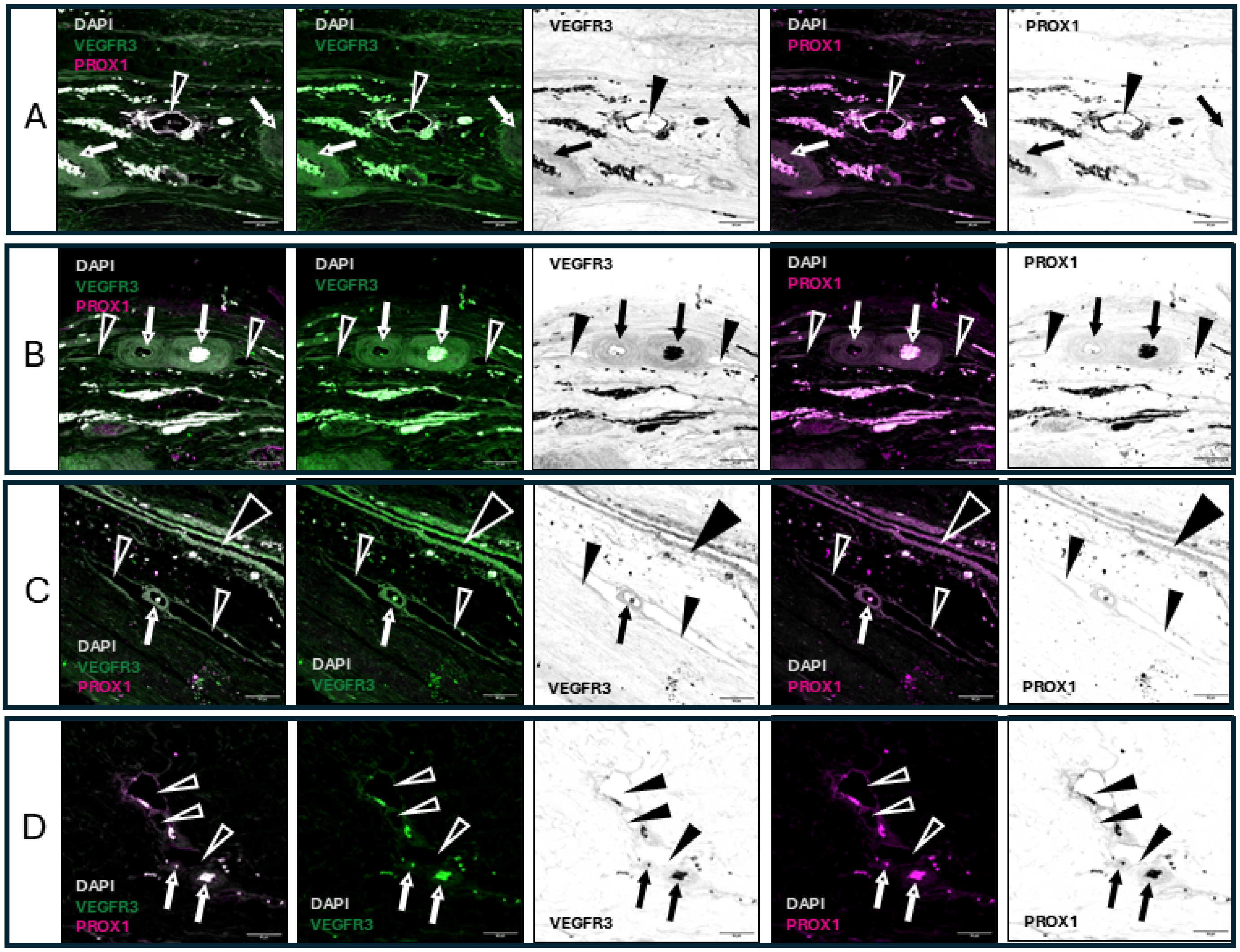

Figure S6. Confocal photomicrographs of representative sections of the common dolphin (*Delphinus delphis*) from the parasagittal meninges surrounding the transverse venous sinus (TVS) using immunofluorescent markers. Scale bars represent 50 µm. (A, D) Animal ID: CAHA559; (B, C) Animal ID: CAHA560. (A-D) Confocal images of the transverse venous sinus meninges displaying merged and individual fluorescent markers for vascular endothelial growth factor receptor-3 (VEGFR3) (green) and Prospero

homeobox 1 (Prox-1) (magenta), with DAPI (white) counter labeling for nuclei. (A) A singular initial meningeal lymphatic vessel (small arrowhead) surrounded by blood vasculature (arrows) with strong Prox-1 and VEGFR3 labeling of lymphatic endothelial cells. VEGFR3 displays strong colocalized positive labeling with Prox-1. (B) Initial meningeal lymphatic vessels in longitudinal cross-section (arrowheads) seated on either side of arterioles in cross-section (arrows) with moderate VEGFR3 labeling of lymphatic endothelial cells. (C) Initial meningeal lymphatic vessels in longitudinal cross-section (small arrowheads) seated on either side of an arteriole (arrow) with moderate VEGFR3 labeling of lymphatic endothelial cells. A larger, pre-collecting (large arrowhead) meningeal lymphatic vessel sits directly above in the connective tissue of the dura, demonstrating strong VEGFR3 labeling of lymphatic endothelial cells with moderate Prox-1 labeling. (D) A chain of meningeal lymphatic vessels (arrowheads) is seen in close association with blood vessels (arrows) demonstrating strong Prox-1 labeling with moderate VEGFR3 labeling. Representative confocal images (individual and merged) demonstrating and differentiating the presence and localization of blood and lymphatic vessels using immunofluorescent markers for vascular endothelial growth factor receptor-3 (VEGFR3) (green) and Prospero homeobox 1 (Prox-1) (magenta), with DAPI (white) counter labeling for nuclei. Note that red blood cells are auto-fluorescent in these images.

Table Supplemental 1. Computed tomography scanning parameters for the common dolphin (*Delphinus delphis*) cranial venous vasculature examination. Animal ID: IFAW12-364. Scanning parameters used for computed tomography scanning and reconstruction of cranial venous vasculature to investigate the superior sagittal sinus and transverse sinus.

CT Parameter	Setting
Slice thickness	1 mm
KVP	120
Data collection diameter	500
Reconstruction diameter	404
Distance SRC (detector)	1040
Distance SRC (patient)	570
Rotation direction	Clockwise
Exposure time	500
